# Modern Sociology

**Published:** 1898-10-01

**Authors:** 


### MODERN SOCIOLOGY.

### PEOPLE'S BANKS.—II.

### The Raffeisen Loan Banks.

Slightly earlier in point of origin, but much slower in point of development, than the "credit associations" of Schultz are the "loan banks" of Herr Raffeisen. The first of these was begun in a small town called Flammersfeld in 1848; but in 1868 there were only four similar associations, all, like the parent one, of small size. This limitation of size is, indeed, an integral part of the organisation. Raffeisen's idea was to interest a whole neighbourhood, rich and poor, in the bank. It was necessary, indeed, that all classes should be members, for there is no share capital in the Raffeisen scheme. As the first loans to the poorer members must come from the richer, it is essential to interest the latter in the working, as they are in the financing, of the institution. The richer members, having the larger liability, are, by common consent, the unpaid directors of the concern. There are no shares, no dividends. At least, such was the original scheme; but as the banking law of Germany now insists on these, the members of the Raffeisen banks meet them by making shares of 10 marks, payable in instalments, and putting all the dividends to reserve, or, when that has grown large enough, voting them away to some scheme or institution of public usefulness.

The Raffeisen banks are meant specially for the poorest of the agricultural classes, and the two outstanding features are long credit and loans on personal security. It was therefore essential that the borrower should be known to the lender at least by repute, and to this end a small circle is of importance. Nobody makes a profit out of the business of lending, and the lender, however kindly disposed, will therefore take no risks. He wants to be sure that his money is secure. Therefore, the improvident man, who borrows from Peter to pay Paul, is not likely to get anything from a Raffeisen bank; his record is against him, and from his neighbours he cannot conceal what that record is. On the other hand, a hard-working and careful man can obtain a loan for a long period on his own personal security. Loans for six months for the purchase of seed are common; loans for two, three, and even five years, to meet the cost of agricultural improvements, by no means the reverse. But a strict supervision is kept over the borrower, for whose benefit alone the bank exists. One safeguard is that when he asks for a loan he must state specifically for what purpose he requires it, and to that purpose he must keep. A loan is not granted to pay miscellaneous debts, but for purposes which must be shown to be economically productive. It must be kept in mind that these banks are founded on the principle of unlimited liability. The apparent danger of this principle has proved the safeguard of the Raffeisen banks; for the directors, knowing the extent of their possible loss in the case of the borrower's default, have been the more careful in granting loans. In this way unlimited liability, dangerous as it sounds, really acts as a check on recklessness instead of encouraging it. Unlimited liability becomes dangerous only when one set of people are liable for the acts of another; but when shareholders and directors are the same person its influence is all the other way. It is here that the advantage of a small association comes in. Practically speaking, every member of a bank can have his eye upon every borrower, and if he knows him to be idle or incompetent, no scruples of politeness will keep him from refusing a loan, if he thinks there is a chance of default. It was to secure this constant surveillance of each by all that Raffeisen recommended that the membership of a bank should be limited to inhabitants of one parish, if it was of fair size; and that even if one or two parishes were joined, they should all be close enough to permit of this intimate acquaintance with the affairs of possible borrowers. He considered that a district with four hundred inhabitants was large enough to support a bank; but he insisted that among the members there should be a sprinkling of the richer classes, not only because they were possessed of capital, but because they had, as a rule, some experience in business ways. So well has the system worked under these safeguards that it is the proud boast of the Raffeisen banks that neither lender nor borrower has ever lost a penny through them. A fair proof of the confidence which they have inspired is the extent to which outsiders offer them deposits. Even in 1866 and 1870, the great war years, when German credit was most sorely tested, and deposits were withdrawn from other banks, they poured into the Raffeisen ones. Ordinary depositors receive 3½ per cent. for their money; while on deposits for a fixed period a slightly higher interest is paid. The average charge for loans is 4½ per cent.

The Raffeisen banks grew slowly at first. The first was founded in 1849, and twenty years after they numbered only four. But their merits had been proved during that period, and since then they have multiplied fast, till they now number about a thousand. Most of these do but a small amount of business; but, then, their expenses are so small that they can afford to deal with small things. The only paid official of each is the cashier, and his remuneration is usually a very modest one. No application for a loan is too insignificant to be considered on its merits. A case is given of an itinerant pastry-cook, who paid £3 a year for the hire of the barrow in which he hawked his wares. The bank advanced him money to buy a barrow. Within a year he had repaid the loan and interest, and henceforth was able to save the sum which he had spent on the hire. A bank such as this really enables a poor man to save money, which without such accommodation he would probably never do.

The moral effect of these Raffeisen banks is said to be admirable. Mr. Henry W. Wolff, in an interesting volume on the subject (Longmans, 1893), says: "People soon find out the value of a cheap lending institution, when they see their neighbours regularly employing it. Once they are made to understand that membership is altogether dependent upon their good character and good conduct, and its continuance upon their perseverance in such virtues, it is astonishing how fast the drunkard forsakes his sottish ways, the spendthrift his extravagance, how fast the idle becomes industrious, the quarrelsome man peaceful, and the reckless careful." In fact, the improvement in the condition of the poor in those places where they have been established seems fully to justify the boast of a recent eulogist, that "the setting up of Raffeisen associations means the pulling down of workhouses."

